# Stem Cells and Cellular Origins of Mammary Gland: Updates in Rationale, Controversies, and Cancer Relevance

**DOI:** 10.1155/2019/4247168

**Published:** 2019-01-08

**Authors:** Jiaojiao Zhou, Qishan Chen, Yiheng Zou, Shu Zheng, Yiding Chen

**Affiliations:** ^1^Department of Surgical Oncology, the Second Affiliated Hospital, Zhejiang University School of Medicine, 88 Jie-Fang Rd, Hangzhou, Zhejiang 310009, China; ^2^The Key Laboratory of Cancer Prevention and Intervention, China National Ministry of Education, 88 Jie-Fang Rd, Hangzhou, Zhejiang 310009, China; ^3^Department of Cardiology, the First Affiliated Hospital, Zhejiang University School of Medicine, 79 Qing-Chun Rd, Hangzhou, Zhejiang 310003, China; ^4^Hangzhou Medical College, 481 Bin-Wen Rd, Hangzhou, Zhejiang 310000, China

## Abstract

Evidences have supported the pivotal roles of stem cells in mammary gland development. Many molecular markers have been identified to characterize mammary stem cells. Cellular fate mapping of mammary stem cells by lineage tracing has put unprecedented insights into the mammary stem cell biology, which identified two subtypes of mammary stem cells, including unipotent and multipotent, which specifically differentiate to luminal or basal cells. The emerging single-cell sequencing profiles have given a more comprehensive understanding on the cellular hierarchy and lineage signatures of mammary epithelium. Besides, the stem cell niche worked as an essential regulator in sustaining the functions of mammary stem cells. In this review, we provide an overview of the characteristics of mammary stem cells. The cellular origins of mammary gland are discussed to understand the stem cell heterogeneity and their diverse differentiations. Importantly, current studies suggested that the breast cancer stem cells may originate from the mammary stem cells after specific mutations, indicating their close relationships. Here, we also outline the recent advances and controversies in the cancer relevance of mammary stem cells.

## 1. Background

Stem cells are a group of undifferentiated cells, possessing two essential properties: the ability to maintain long-term self-renewal and capacity to differentiate into specialized cell lineages [1]. Mammary gland is a unique exocrine glandular organ, undergoing cyclic expansions during menstrual cycles and dramatic changes in structure and function during pregnancy, lactation, and involution [2]. Mammary stem cells (MaSCs), which defined as the stem cells existing in mammary gland, are essential for maintaining mammary homeostasis and repair. Unlike most other mammalian organs that developed in embryonic phase, mammary gland develops greatly postnatally, further emphasizing the pivotal roles of the adult stem and progenitor cells on mammary gland.

Here, we reviewed current advances of studies in stem cells and cellular origins of mammary gland, including MaSCs in mammary gland development, molecular markers of MaSCs, cellular fate mapping of MaSCs by lineage tracing, and stem cell niche as a regulator in sustaining MaSC function. Moreover, considering the significantly tumorigenic roles of stem cells in cancer, we also discussed about the relationships between MaSCs and breast cancer stem cells (BCSCs), as well as the potential regulatory mechanisms of the MaSCs that deviated in breast cancer.

## 2. MaSCs and Mammary Gland Development

**Figure 1:**
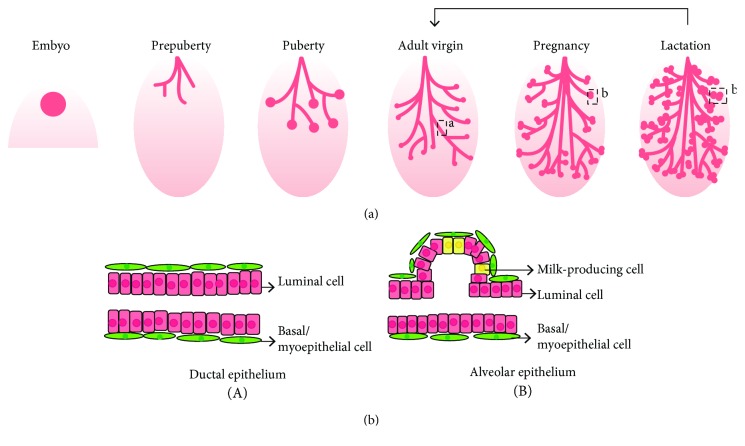
(a) The postnatal mammary gland development is multistage. (b) Two distinct phenotypes of mammary epithelium in different developmental stages: the ductal (A) and alveolar (B) epithelium, both bilayered, with inner layer of luminal cells and outer layer of myoepithelial/basal cells. There are also milk-producing cells in the inner layer of alveolar epithelium.

The mammary gland undergoing extensive development after birth throughout puberty, pregnancy, lactation, and involution (Figure 1(a)) is a remarkably adaptive organ whose development is closely regulated by the steroid and peptide hormones [3]. Human mammary gland is a branching tree-like structure, composed of the epithelium and surrounding stroma [4]. The bilayered mammary epithelium comprises inner layer of luminal cells and outer layer of basal or myoepithelial cells (basal/myoepithelial cells) [5]. The phenotype of epithelium is distinct in mammary development, including ductal phenotype in puberty and adult virgin (Figure 1(b), A) and alveolar phenotype in pregnancy and lactation (Figure 1(b), B) [3]. Interestingly, the alveolar epithelium undergoes a significant amount of remodeling during each pregnant cycle [3]. Starting in pregnancy, the alveolar epithelium proliferates and differentiates rapidly in response to circulating hormonal changes [2]. Then in lactation, the luminal cells synthesize and secrete the milk, while the surrounding myoepithelial cells contract to deliver the milk. Last, during weaning, the expanded compartments of the mammary epithelium undergo apoptosis with the extracellular matrix remodeling [6]. The profound capacity for alveolar renewal in each subsequent pregnancy makes people believe the existence of long-lived mammary stem cells (MaSCs). A number of transplantation experiments [7–9] have proved that fragments of mammary tissue could reproduce the entire epithelial ductal trees in the clear fat pad of recipient mice. Moreover, the emerging single-cell RNA profiles of mammary epithelium further supported the existence of MaSCs and revealed their dynamic differentiation [10, 11].

The MaSCs have been proposed as the cells that can renew themselves and give rise to the epithelial precursor cells (EPCs) [9], which destined for either luminal or basal/myoepithelial cells [12]. Over the past two decades, clonogenic assays [13], transplantation [14], and lineage tracing experiments [15] have been mainly used to evaluate the renewal and differentiation potential of MaSCs. In particular, these studies of mammary gland development have shed light on the identification of specific surface markers [16] and the cellular fate mapping of MaSCs and EPCs [15, 17, 18], as well as the regulation of mammary cellular hierarchy [2]. To some extent, interest in MaSCs was also greatly stimulated by their potential role in breast carcinogenesis.

### 2.1. Molecular Markers of MaSCs

The mammary epithelium undergoes dynamic cycles of growth and involution throughout life, displaying dramatic regenerative potential. The mammary fat pad transplantation assays over the past seventy years have provided the convincing proof of the existence of MaSCs and allowed the recent prospective isolation of MaSCs. The “gold-standard” transplantation assay for the mammary gland reconstitution in mice was established by Deome et al. [19] in 1959. Using transplantation assays, it was demonstrated that mammary epithelium could be regenerated by implanted small fragments [7, 8] or cell suspension [20]. In 1998, Kordon and Smith [9] showed that the entire mammary epithelium was recapitulated by a single stem cell, which was further verified by Shackleton et al. [16] in 2006, describing that a single self-renewing Lin^−^CD29^hi^CD24^+^ cell repopulated a completely functional mammary gland.

Classically, MaSCs populations were identified and isolated using fluorescence-activated cell sorting (FACS), followed by the examination of their reconstitution capacity by transplantation assays *in vivo* [16, 21–29]. Many MaSC specific cellular markers have been used (Table 1). In FACS, markers of CD45, Ter119, and CD31 (also named as Lin) were usually used to exclude the hematopoietic and endothelial cells first. CD24 (heat-stable antigen), CD29 (*β*1-integrin), and CD49f (*α*6-integrin) were commonly used as the MaSC specific markers across studies [16, 21, 23–29]. Other markers such as Lrp5/6 [22], Axin2 [23], CD1d [25], Lgr5 [27], and Procr [29] were reported to identify MaSCs in a single study, respectively. It is notable that these markers are refined from the Wnt signaling pathway (Table 1), the pathway that proved to be instrumental for MaSC self-renewal and long-term expansion [30]. Recently, Zeng et al. [31] reported that the Ccnys-deficient mammary cells failed to reconstitute, revealing the importance of the Ccnys-mediated mitotic Wnt signaling in MaSCs and mammary gland development. Besides, *α*-SMA^+^ and Myh11^+^were also recognized as the MaSC markers, which *α*-SMA^+^ and Myh11^+^myoepithelial cells have the mammary repopulating unit (MRU) capacity [28].

MRU was first defined by Stingl et al. [21], referring to the cell populations with the ability to regenerate new mammary tissue on transplant at limiting dilutions *in vivo*. In MaSC studies, the MRU frequency is a significant index to evaluate the mammary reconstitution capacity of the cells. However, it is obvious that the MaSC markers and MRU frequency were various from study to study (Table 1). One plausible explanation can be the methodological variations, including different donor mice age, transplant conditions, and subtle technical differences in harvesting and processing the MaSC populations [32]. Intrinsically, a more probable explanation is that the sorted cells with MRU capacity were just restricted subsets of MaSCs across different studies, while the different subsets of MaSCs may have distinct expression markers and give rise to the MRU frequency diversely.

Although these studies have given massive information about markers and regenerative features of MaSCs, the exact identity of mammary stem cells is still controversial. Meanwhile, there are many doubts about the transplantation assay, arguing with the artificiality of the MRU *in vivo*.

## 3. Cellular Fate Mapping of MaSCs by Lineage Tracing

**Figure 2:**
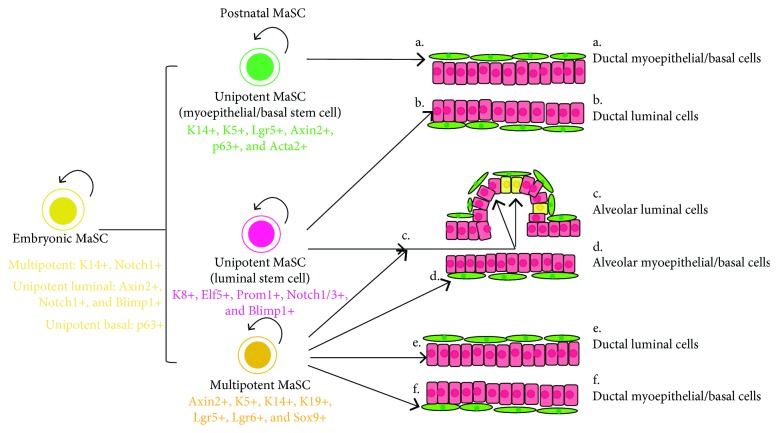
Cellular fate mapping of MaSCs by lineage tracing *in vivo*. A simplified schematic depicts the existence of unipotent and multipotent MaSCs characterized with distinct cellular markers (embryonic MaSCs: K14+, Axin2+, Notch1, Blimp1+, and p63+; postnatal MaSCs for unipotent basal lineage: K14+, K5+, Lgr5+, Axin2+, p63+, and Acta2+; postnatal MaSCs for unipotent luminal lineage: K8+, Elf5, Prom1+, Notch1+, Notch3+, and Blimp1+; multipotent postnatal MaSCs: Axin2+, K5+, K14+, K19+, Lgr5+, Lgr6+, and Sox9+) and their distinct differentiations.

Studies have indicated the presence of different types of MaSCs existing in mammary gland, including the multipotent and unipotent MaSCs. The multipotent MaSCs are able to differentiate to either myoepithelial or luminal lineage mammary cells, while the unipotent MaSCs feature the lineage-restricted differentiation potential (Figure 2). To further investigate the differentiation and cell fate of the MaSCs, lineage tracing is increasingly employed in tracking MaSCs and their progeny in situ.

Genetic lineage-tracing technique is a powerful tool for mapping the cellular fate of stem cells, because it can directly observe all the progeny of a single stem cell under physiological or pathological conditions in mouse model [33]. In the technique of lineage tracing, a recombinase enzyme is expressed in a cell- or tissue-specific manner to specifically activate the expression of a conditional reporter gene, which can make permanent genetic labeling of all progeny of the marked cells [34]. At present, *Cre-loxP* system [35] is the preferred approach of genetic lineage tracing in mice, owing to its high recombination efficiency. In the lineage tracing using *Cre-loxP*, Cre recombinase is expressed under the cell-specific promoter, and specifically activates the reporter in the cells that express the promoter, by removing the STOP cassette in *loxP*-STOP-*loxP* sequence. To make the temporal and spatial control of Cre activity, CreER is recently used in lineage tracing, which the Cre activity is inducible via ER ligand tamoxifen.

Several important lineage-tracing studies of mammary gland have emerged in recent years (Table 2), in which the keratin family was selected as the classic markers for labeling the stem cells in these lineage-tracing studies. Van Keymeulen et al. [15] found that embryonic K14^+^ (keratin14) stem cells were multipotent, while postnatal K14^+^ stem cells were unipotent which only contributed to the myoepithelial lineage during puberty, adult life, and pregnancy. They also found that two other putative stem cell markers, K5^+^ (keratin5) and Lgr5^+^, preferentially labelled the myoepithelial stem cells [15]. For the luminal stem cell markers, their lineage-tracing assay showed that the K8^+^ (keratin8) cells contained the unipotent luminal stem cells, which differentiated into luminal and milk-producing cells [15]. Although the K18^+^ (keratin18) cells also only labelled the luminal cells, no clonal expansion of K18^+^ luminal cells was observed during puberty, virgin, and pregnancy, which indicated the K18^+^ cells as more committed luminal cells [15]. In conclusion, Van Keymeulen's study illustrated that the unipotent luminal and myoepithelial stem cells, respectively, controlled each lineage throughout the mammary development. Rios et al.'s study [36], however, showed the existence of multipotent stem cells during the mammary development. They depicted that the K5, K14, or Lgr5 targeted long-lived stem cells were multipotent, which contributed to the expansion of both luminal and myoepithelial lineages in the pubertal and adult mammary gland, as well as the alveologenesis during pregnancy. However, Elf5^+^ (E74-like factor 5) stem cells were found to be unipotent, which only contributed to the luminal lineage through puberty and into adulthood. Besides, the Elf5^+^ cells also contributed to the generation of alveolar cells in pregnancy. Taken together, the discrepancies between the two studies, such as the different differentiation potency of K14^+^, K5^+^, and Lgr5^+^ cells, can be partially explained by the different lineage-tracing mouse models (Table 2), relating to different labeling efficiency. It is also possibly because different concentrations of the induction agent (tamoxifen) resulted in the different labelling intensity [37]. Actually, in *Cre-loxP* system, the commonly used induction agent tamoxifen may influence the mammary stem cell behaviors [37, 38]. Wuidart et al. [39] further assessed the lineage relationship and stem cell fate in mammary gland, by quantitative lineage-tracing strategies. Stem cells labeled Lgr5^+^ or Lgr6^+^ targeted about 60% of basal cells and 40% of luminal cells, while stem cells labeled K19^+^ or Sox9^+^ targeted more than 95% of luminal cells and less than 5% of basal cells. And for K14^+^ stem cells, they targeted initially and independently unipotent luminal and basal cells in mammary gland. However, the mathematical modeling by Wuidart et al. has been queried in interpreting the image data and quantifying model parameters [40], as the proteolytic digestion used for tissue processing can destruct the basal lamina and profoundly change the morphology of epithelial cells and their physical interaction with luminal cells. Thus, care must be taken in such statistical models of lineage tracing. More extensive images derived from refined genetically engineered mice that allow different populations to be marked are needed for giving more precise evaluation.

Besides the keratin family, Notch family including Notch1+, Notch2+, and Notch3+ have also been found to mark MaSCs *in vivo* (Table 2), corresponding with that Notch signaling pathway was greatly implicated in mammary gland development. For Notch1, Rodilla et al. [41] found that Notch1 targeted multipotent stem cells in the embryonic mammary bud but restricted their lineage potential to ER- luminal lineage postnatally. Later, Lilja et al. [42] further reported that Notch1 activation would lock multipotent stem cells into a luminal unipotent cell fate during early mammary embryogenesis and then specially dictated ER- luminal cell fate postnatally. By using Notch2-specific genetic labeling, Sale et al. [43] uncovered the existence of distinct Notch2+ progenitors that represent two previously unrecognized mammary epithelial cell lineages, which they termed S (small) and L (large) cells. And the S and L cells are morphologically, topologically, genetically, developmentally, and functionally distinct from classical luminal and myoepithelial cells. Lafkas et al. [44] elucidated that Notch3^+^ cells were a highly clonogenic and transiently quiescent luminal progenitor population that gives rise to a ductal lineage.

Furthermore, many other notable lineage-tracing studies emerged in recent years (Table 2), with targeted stem cells or progenitors expressing Axin2 [18], Acta2 [28], WAP [45], Procr [29], Lgr5 [15, 36, 39], Lgr6 [39], Sox9 [39, 46], prominin1 [46], p63 [47, 48], ER [49], and Blimp1 [50]. These studies not only supported the coexistence of different multipotent and unipotent stem cells in the mammary epithelium but also revealed the dynamic developmental fate of mammary stem cells.

Notably and intriguingly, recent studies revised previous model of cellular hierarchy of luminal cells and provided solid evidence that ER^−^ and ER^+^ luminal cells were even maintained by distinct stem cells (Table 2). Until now, studies have proved that Wap+ [45], Sox9+ [46], Blimp1+ [50], and Notch1+ [41, 42] stem cells contributed to ER- luminal lineage cells postnatally, while Prom1+ [46] and ER+ [49] stem cells restricted to differentiate into ER+ luminal lineage. These findings revised the understandings of mammary epithelial cell hierarchy and further supported that ER^−^ and ER^+^ luminal cells are two independent lineages.

Thus, studies still presented unclear results, although the genetic lineage tracing has put unprecedented insights into the mammary stem cell biology. More studies are needed to determine the relationships between all these mammary stem cell populations of different markers' expression. Certainly, more studies applying lineage-tracing technique are urged to enrich the comprehensive understandings on cellular origins of mammary epithelium.

## 4. Lineage Signatures of Mammary Epithelium by Single-Cell RNA-Seq

The comprehensive single-cell transcriptomes are recently used as a powerful tool to understand cellular hierarchy and lineage relationships. Two recent studies [10, 11] that used single-cell RNA sequencing have supported the existence of MaSCs and mapped the cellular dynamics of mammary epithelium at different developmental stages.

In the study by Pal et al. [10], they newly identified a mixed-lineage or “lineage-primed” cluster among basal cells which may precede commitment to the luminal lineage during puberty, adulthood, and pregnancy. These cells expressed both core basal and luminal genes, such as Acta2, Krt14, Cxcl4, Myh11, Areg, Elf5, Krt19, and Csn2. An early progenitor subset (Lum Int) marked by CD55 was also depicted in their study, lying between luminal progenitor and mature ductal/alveolar cells, with expression of Jund, Irx5, Sox4, and Igfbp2. In the study by Bach et al. [11], they analyzed 23,184 cells across nulliparous, mid gestation, lactation, and post involution and identified 15 distinct clusters of mammary epithelial cells. In the luminal compartment, both the hormone sensing and not subgroup possessed clusters that expressed progenitor markers (e.g., Aldh1a3, CD14, Kit), while the basal compartment also contained a cluster of “stem-like” cells that expressed high levels of Procr, Gng11, and Zeb2. In summary, the data of single-cell transcriptomes provides us an unbiased view of mammary gland development and unmasks the lineage signature of mammary epithelium at a high cellular resolution. More single-cell sequencing profiles at different developmental time-points are needed to give a more comprehensive understanding on the molecular networks that drive specification and differentiation in mammary gland.

## 5. The Stem Cell Niche as a Regulator in Sustaining MaSC Function

MaSCs are located in the specific microenvironment which is called as MaSC “niche” [51]. Paracrine factors and extracellular matrix (ECM) were the pivotal MaSC niche elements in regulating MaSC maintenance and differentiation [52]. Aberrant regulation may increase the opportunity for accumulation of oncogenic mutations in the self-renewing MaSCs, eventually leading to the neoplastic progression.

Mammary gland is one of the main target organs for steroid hormone, including estrogen, progesterone, and prolactin. These steroid hormones play important roles in controlling ductal outgrowth and alveolar expansion. Both global [53] and conditional ER*α* knockout mice [54] revealed the essential requirement of ER*α* for epithelial proliferation and morphogenesis in mammary development. Yet, substantial evidence has showed that steroid hormones exert their effects on MaSCs through paracrine signaling. At first, Asselin-Labat et al. [55] found that the expression of ER*α* and PR were high in luminal cell-enriched (CD24^+^CD29^lo^) population, indicating the importance of luminal cells in ER*α* and PR signaling. Later, they demonstrated that MaSCs were highly responsive to the steroid hormone via paracrine signaling from the RANK (also called Tnfrsf11a) ligand produced by luminal cells [56]. It is also demonstrated by Joshi et al. [57] that progesterone propelled MaSC expansion *in vivo* during the reproductive cycle, which acted mitogenic effect on MaSCs through paracrine signaling from the RANK ligand and Wnt4 produced by luminal cells. Besides, studies by Lee et al. [58] showed that the paracrine signaling of progesterone-RANK ligand exerted effects on Elf5 expression in CD61^+^ (integrin *β*3) luminal progenitor cells and their consequent differentiation. Moreover, novel mediator such as Rspo1 (R-spondins1) has been recently found to be implicated in promoting MaSC self-renewal through the synergy action with Wnt4 [59]. Taken together, all these studies suggested that the steroid hormones normally regulate the MaSCs, probably through the paracrine signals from the ER^+^ luminal cells.

It is widely believed that there are MaSCs localized in the basal layer of adult mammary epithelium, which directly interact with the ECM. The mammary basal cells were found with high expression of integrins [60], which are the major class of receptors for ECM [61]. As we know, integrins such as *α*6 and *β*1-integrins (CD49f and CD29) have already been commonly used as the markers to purify MaSCs, indicating their potential roles in regulating MaSCs. Taddei et al. [62] found that *β*1 integrin deletion from the basal cells abolished the MaSC maintenance and mammary morphogenesis, validating their essential roles in mediating the interactions between ECM and MaSCs contained basal cells. Besides, MMPs (matrix metalloproteinases), which are the essential microenvironmental proteases in degrading and remodeling ECM, were found to play an important role in regulating MaSC functions. MMP3 produced in the vicinity of mammary epithelium could promote MaSC function by binding and activating Wnt5b [63]. Other MMPs such as MMP14 [64] were also proved to be important in mammary development. Thus, there is no doubt that MaSC niche plays a crucial role in regulating MaSCs, and more underlying mechanisms need to be further investigated.

## 6. Relationships between MaSCs and BCSCs

MaSCs and breast cancer stem cells (BCSCs) are distinct with each other but also have much in common. To some extent, the hypothesis of “cancer stem cell” is a derivative of the “normal stem cell” concept [65], stating that cancer cell populations are hierarchically developed, with cancer stem cells at the apex of the hierarchy [66]. Indeed, BCSCs often share features with MaSCs; for instance, they share the same cellular markers such as CD29 [67], CD49f [67], Lgr5 [68], Procr [69], and CD61 [70]. The understanding of MaSC roles in normal breast is crucial to elucidate the critical functions of BCSCs in breast cancer.

However, do BCSCs originate from MaSCs and what is the potential mechanism? One hypothesis is that the routine self-renewal and expansion of MaSCs increase the opportunity for the accumulation of oncogenic mutations and lead to the altered control of differentiation and proliferation, which may predispose to breast cancer. Convincing evidence in mouse models suggested the potential roles of MaSCs in tumorigenesis. The transcriptome analyses revealed that breast tumors arising from MMTV-Wnt-1 and p53^−/−^ mice were enriched for MaSC-subset (CD29^hi^CD24^lo^CD61^+^) genes, whereas tumors of MMTV-Neu and MMTV-PyMT mice were enriched for luminal progenitor subset (CD29^lo^CD24^+^CD61^+^) genes [71]. Wnt signaling may play an important role in the transit from MaSCs to BCSCs [72]. It was illustrated that the Wnt-1-induced mammary tumor expanded an epithelial subpopulation, which expressed MaSC markers such as K6 (keratin 6) and Sca-1, indicating that the ectopic Wnt pathway may target MaSCs for tumorigenesis [73]. Importantly, recent studies by Koren et al. [74] and Van Keymeulen et al. [75] strongly support the statement on reprogramming differentiated cells towards cancer stem cells in breast cancer, by using oncogenic Pik3CA^H1047R^ mutant mouse model. Both of the studies unraveled a key effect of Pik3CA^H1047R^ on mammary cell fate at the early stage of tumor initiation, which activated a multipotent genetic program [74, 75].

In brief, BCSCs may derive from MaSCs or early stem cell progenitors through the accumulation of oncogenic mutations, but direct evidence for this oncogenic evolution hypothesis is still less well established. Moreover, it is also possible that BCSCs could originate from more differentiated cells but not MaSC population [76]. Much more precise studies are still needed.

## 7. Conclusions and Perspectives

In the recent two decades, impressive advances have been witnessed in understanding the mammary gland development, in which MaSC hypothesis provided very important models. A variety of cellular markers and specific regulatory signalings were identified in MaSCs, as well as some overlap observed. In mammary gland, cellular fate mappings of MaSCs, by lineage tracing, identified the unipotent and multipotent MaSCs, which specifically differentiate to luminal or basal cells. Certainly, the molecular portraits of MaSCs were greatly influenced by the stem cell niche. Given the potential role of MaSCs in breast carcinogenesis, current studies suggested that BCSCs may originate from the MaSCs after specific mutations. However, indubitably, much cognition for MaSCs is still obscure, such as the following: is there a distinct and universal molecular signature for MaSCs? Is there a hierarchical relationship between multipotent and unipotent MaSCs? How does the multipotent MaSCs differentiate into the restricted luminal or basal lineage? Within the embryonic or postnatal MaSCs, what is the relationship among the MaSCs of different marker's expression? How does the stem cell niche cooperatively or competitively regulate the MaSCs functions? More precise evidence is required for the transition potency of MaSCs into BCSCs or their potential oncogenic capacity. In a word, challenge is still ahead, but the comprehensive understandings of stem cells and cellular origins in mammary gland have already and will continue to help us to intimately know the biological and pathologic development of mammary gland and overcome the stubborn breast cancer ultimately.

## Figures and Tables

**Table 1:** Markers used to identify MaSCs in different studies.

Study	MSC markers by FACS	Markers' annotations	Gland reconstitution donor	MRU frequency/gland outgrowth frequency
Stingl et al. [21]	CD45^−^Ter119^−^CD31^−^ CD140a^−^CD24^med^CD49f^hi^	CD45^−^ and Ter119^−^: to exclude the haematopoietic cells CD31^−^: to exclude the endothelial cells CD140a^−^: to exclude the stromal cells CD24: heat-stable antigen, expressed by the apical plasma membrane of the luminal cells CD49f: *α*6-integrin, expressed by epidermal stem cells and human mammary colony forming cells	8-14-week-old virgin FVB, C57Bl/6 mice (in 2%FBS)	1 per 60 CD24^med^CD49f^hi^ (from FVB mice) 1 per 90 CD24^med^CD49f^hi^ (from C57Bl/6 mice)

Shackleton et al. [16]	CD45^−^CD31^−^TER119^−^ (Lin^−^) CD24^+^CD29^hi^	CD24: heat-stable antigen, also expressed by neural stem cells and human breast cancer cells CD29: *β*1-integrin, expressed in skin stem cells	8-week-old Rosa-26 mice (with LacZ transgene) (in 50% FBS)	1 per 64 Lin^−^CD24^+^CD29^hi^

Badders et al. [22]	CD45^−^CD31^−^Lrp5^+^	Lrp5: Wnt coreceptor, required for mammary ductal stem cell activity and Wnt1-induced tumorigenesis	10-week-old virgin BALB/c mice (in Matrigel)	1 per 485 Lrp5^hi^ 1 per 142 CD24^+^CD29^hi^

Zeng and Nusse [23]	CD31^−^CD45^−^Ter119^−^ CD24^+^CD29^hi^Axin2^+^	Axin2: Wnt/*β*-catenin target gene in canonical Wnt signal transduction pathway	8-12-week-old Axin2-lacZ reporter mice (in 50% Matrigel or 50% serum)	3 per 5 glands Lin^−^CD24^+^CD29^hi^Axin2^+^ (from 500 cells) with serum 1 per 5 gland Lin^−^CD24^+^CD29^hi^Axin2^+^ (from 100 cells) with serum 6 per 8 glands Lin^−^CD24^+^CD29^hi^Axin2^+^ (from 100 cells) with Matrigel 11 per 16 glands Lin^−^CD24^+^CD29^hi^Axin2^+^ (from 50 cells) with Matrigel

Spike et al. [24]	CD31^−^CD45^−^Ter119^−^ CD24^med^CD49f^hi^		Fetal (E18.5) or adult CD1 mice (with/without Matrigel)	1 per 14 fetal CD24^hi^CD49f^hi^ with Matrigel 1 per 50 adult CD24^med^ CD49f^hi^ with Matrigel

Plaks et al. [27]	CD31^−^CD45^−^Ter119^−^(Lin^−^) CD24^+^CD49f^hi^CK14^+^Lgr5^+^	Lgr5: a downstream target of Wnt and a major stem cell marker in the small intestine, colon, stomach, hair follicle, and kidney nephrons CK14: a major marker of mammary myoepithelial/basal cells	7-9-week-old Lgr5-EGFP pubertal mice (in Matrigel with FGF2)	3 per 9 glands Lin^−^CD24^+^CD49f^hi^CK14^+^Lgr5^+^ (from 100 cells) 2 per 12 glands Lin^−^CD24^+^CD49f^hi^CK14^+^Lgr5^+^ (from 50 cells) 5 per 16 glands Lin^−^CD24^+^CD49f^hi^CK14^+^Lgr5^+^ (from 10 cells)

Machado et al. [26]	Lin^−^CD24^+^CD29^hi^ large (>10 *μ*m diameter)		8-12-week-old FVB.Cg-Tg(CAG-EGFP)B5Nagy/J female mice (in Matrigel)	1 per 66 Lin^−^CD24^+^CD29^hi^, >10 *μ*m diameter 1 per 132 Lin^−^CD24^+^CD29^hi^ 1 per 237 Lin^−^, >10 *μ*m diameter

dos Santos et al. [25]	CD31^−^CD45^−^Ter119^−^(Lin^−^) CD24^+^CD29^hi^CD49f^hi^CD1d^+^	CD1d: a glycoprotein expressed on the surface of various mouse and human antigen-presenting cells	6-10-week-old H2b-GFP transgenic virgin mice (in 50% Matrigel)	1 per 8 Lin^−^CD24^+^CD29^hi^CD1d^+^1 per 44 Lin^−^CD24^+^CD29^hi^

Prater et al. [28]	CD31^−^CD45^−^Ter119^−^ CD49f^hi^EpCAM^hi^*α*SMA^+^or Myh11^+^	EpCAM: epithelial cell adhesion molecule; *α*SMA and Myh11 are functional markers of myoepithelial cells and enhance contractile force generation during lactation	10-14-week-old C57BL/6J, Acta2–GFP and Myh11–Cre–GFP;Rosa26LacZ virgin mice (in 25% Matrigel)	1 per 57 Basal EpCAM^hi^ 1 per 93 Basal *α*SMA^hi^ 1 per 67 Basal Myh11^+^

Wang et al. [29]	Lin^−^CD24^+^CD29^hi^Procr^+^	Procr: a novel Wnt target, a protein C receptor, functions in anticoagulation, inflammation, and haematopoiesis	8-week-old CD1 mice (in 50% Matrigel and 20% FBS)	1 per 68 CD24^+^ CD29^hi^ 1 per 12 CD24^+^ CD29^hi^Procr^+^

Zeng et al. [31]	Lin^−^CD24^+^CD29^+^Ccnys^−^ lost the basal stem cell function in regeneration	Ccnys: Ccny and paralogue Ccnyl1, essential in Wnt signaling activity for maintaining the developmental potential of dividing MSCs; expression of Ccnyl1 and Axin2 overlapped in pubertal mammary gland	8-12-week-old transgenic mice (in 50% Matrigel and 20% FBS)	1 per 5024 Ccny^+/−^; Ccnyl1^+/lacZ^ + scramble-shRNA (loss of 2 copies) 1 per 13355 Ccny^−/−^; Ccnyl1^+/lacZ^ + scramble-shRNA (loss of 3 copies) None Ccny^−/−^; Ccnyl1^+/lacZ^ + Ccnyl1-shRNA (Ccnys depleted)

MRU: mammary repopulating unit; CD45: protein tyrosine phosphatase receptor type C; Ter119: lymphocyte antigen 76; CD31: platelet/endothelial cell adhesion molecule 1; CD140: platelet-derived growth factor receptor; CD49f: *α*6-integrin; CD29: *β*1-integrin; Lrp5: LDL receptor-related protein 5; Lgr5: leucine-rich repeat-containing G protein-coupled receptor 5; EpCAM: epithelial cell adhesion molecule; *α*-SMA: alpha smooth muscle actin; Myh11: smooth muscle myosin, heavy polypeptide 11.

**Table 2:** An overview of lineage-tracing studies defined MaSC markers and their cellular fate using different mouse models.

Study	Marked cells	Cellular fate of the MaSCs	Mouse model
Van Keymeulen et al. [15]	K14+	Embryonic: multipotent	K14-Cre/Rosa-YFP mice K14-rtTA/TetO-Cre/Rosa-YFP mice
	K14+	Postnatal: unipotent (myoepithelial/basal)	K14-rtTA/TetO-Cre/Rosa-YFP mice
	K5+	Postnatal: unipotent (myoepithelial/basal)	K5-CreER/Rosa-YFP mice
	Lgr5+	Postnatal: multipotent (most basal, rare luminal)	Lgr5-GFP-CreER/Rosa-Tomato mice
	K8+	Postnatal: unipotent (luminal)	K8-CreER/Rosa-YFP mice
	K18+	Postnatal: unipotent (luminal)	K18-CreER/Rosa-YFP mice

Van Amerongen et al. [18]	Axin2+	Embryonic: unipotent (luminal)	Axin2^CreERT2/+^;R26R^mTmG/+^ mice Axin2^CreERT2/+^;R26R^lacZ/+^ mice
		Prepubety: unipotent (myoepithelial/basal)	
		Puberty: multipotent	
		Pregnancy: multipotent	

Sale et al. [43]	Notch2+	Postnatal: multipotent (small and large cells) (unrecognized mammary epithelial cell populations)	N2-CreERT2^SAT^/R26R^LacZ^

Lafkas et al. [44]	Notch3+	Postnatal: unipotent (luminal)	N3-CreERT2^SAT^/R26^mTmG^ mice

Rios et al. [36]	Elf5+	Postnatal: unipotent (luminal)	Elf5-rtTA-IRES-GFP mice Elf5-rtTA/TetO-cre/R26R-Confetti mice Elf5-rtTA/TetO-cre/R26R-tdTomato mice
	K5+	Postnatal: multipotent	K5-rtTA-IRES-GFP mice K5-rtTA/TetO-cre/R26R-Confetti mice K5-rtTA/TetO-cre/R26R-tdTomato mice
	K14+	Postnatal: multipotent	K14-creERT2/R26R-Confetti mice
	Lgr5+	Postnatal: multipotent	Lgr5-GFP-IRES-creERT2/R26R-tdTomato mice

Prater et al. [28]	Acta2+	Postnatal: unipotent (basal)	Acta2-Cre-ER^T2^;Rosa26LacZ mice Acta2–Cre–ER^T2^; R26^mTmG^ mice

Rodilla et al. [41]	Notch1+	Embryonic: multipotent Postnatal: unipotent (ER- luminal)	N1Cre^ERT2^R26^mTmG^ mice

Chang et al. [45]	Wap+	Pregnancy: unipotent (ER- luminal)	WAP-Cre;Rosa26-lsl-YFP mice

Wang et al. [29]	Procr+	Postnatal: multipotent	Procr^CreERT2/+^;R26^mTmG/+^ mice Procr^CreERT2/+^;R26^DTA/+^ mice

Wuidart et al. [39]	K14+	Postnatal: multipotent	K14-CreER^T2^/Rosa-Confetti mice
	K19+	Postnatal: multipotent (most luminal, rare basal)	K19-CreER^T^/Rosa-Confetti mice
	Sox9+	Postnatal: multipotent (most luminal, rare basal)	Sox9-CreER^T2^/Rosa-Confetti mice
	Lgr5+	Postnatal: multipotent	Lgr5-CreER^T2^/Rosa-tdTomato mice
	Lgr6+	Postnatal: multipotent	Lgr6-CreER^T2^/Rosa-tdTomato mice

Wang et al. [46]	Sox9+	Postnatal: multipotent (myoepithelial/basal, ER- luminal)	Sox9-CreER^T2^;R26R-tdTomato mice
	Prom1+	Postnatal: unipotent (ER+ luminal)	Prom1-CreER^T2^;R26R-tdTomato mice

Sreekumar et al. [47]	p63+	Postnatal-Cap cells: unipotent (basal)	p63^CreERT2/+^;Rosa^mTmG/+^

Van Keymeulen et al. [49]	ER+	Postnatal: unipotent (ER+ luminal)	ER-rtTA/TetOH2B-GFP mice ER-rtTA/TetOCRE/Rosa-YFP mice

Elias et al. [50]	Blimp1+	Embryonic: unipotent (luminal) Postnatal: unipotent (ER- luminal)	Prdm1Cre^ERT2/+^; R26R^mTmG/+^

Wuidart et al. [48]	p63+	Embryonic: unipotent (basal)	K14rtTA/TetO-Cre/Rosa-Δ Np63-IRES-GFP

Lilja et al. [42]	Notch1+	Embryonic: unipotent (luminal) Postnatal: unipotent (ER- luminal)	N1Cre^ERT2^/R26^mTmG^ mice

K14: keratin14; K5: keratin5; K8: keratin8; K18: keratin18; K19: keratin19; Elf5^+^: E74-like factor 5; Acta2: actin, alpha 2, smooth muscle, aorta; WAP: acidic protein; Procr: protein C receptor; Sox9: SRY-box 9; Lgr6: leucine-rich repeat-containing G protein-coupled receptor 6; Prom1: prominin 1; p63: tumor protein p63; ER: estrogen receptor1; Blimp1: PR/SET domain 1.

## Abbreviations

MaSCs: Mammary stem cells
EPCs: Epithelial precursor cells
CSCs: Cancer stem cells
BCSCs: Breast cancer stem cells
FACS: Fluorescence-activated cell sorting
CD24: Heat-stable antigen
CD29: *β*1-Integrin
CD49f: *α*6-Integrin
MRU: Mammary repopulating unit
CD45: Protein tyrosine phosphatase receptor type C
Ter119: Lymphocyte antigen 76
CD31: Platelet/endothelial cell adhesion molecule 1
CD140: Platelet-derived growth factor receptor
Lrp5: LDL receptor-related protein 5
Lgr5: Leucine-rich repeat-containing G protein-coupled receptor 5
EpCAM: Epithelial cell adhesion molecule
*α*-SMA: Alpha smooth muscle actin
Myh11: Smooth muscle myosin, heavy polypeptide 11
K14: Keratin14
K5: Keratin5
K8: Keratin8
K18: Keratin18
K19: Keratin19
Elf5+: E74-like factor 5
Acta2: Actin, alpha 2, smooth muscle, aorta
WAP: Acidic protein
Procr: Protein C receptor
Sox9: SRY-box 9
Lgr6: Leucine-rich repeat-containing G protein-coupled receptor 6
Prom1: Prominin 1
p63: Tumor protein p63
ER: Estrogen receptor1
Blimp1: PR/SET domain 1
Cxcl4: Platelet factor 4
Areg: Amphiregulin
Csn2: COP9 signalosome subunit 2
CD55: CD55 molecule
Jund: JunD proto-oncogene
Irx5: Iroquois homeobox 5
Sox4: SRY-box 4
Igfbp2: Insulin-like growth factor-binding protein 2
Aldh1a3: Aldehyde dehydrogenase 1 family member A3
CD14: CD14 molecule
Gng11: G protein subunit gamma 11
Zeb2: Zinc finger E-box binding homeobox 2
ECM: Extracellular matrix
CD61^+^: Integrin *β*3
Rspo1: R-spondins1
MMPs: Matrix metalloproteinases
ALDH1: Aldehyde dehydrogenase1.

## References

[1] Morrison S. J., Spradling A. C. (2008). Stem cells and niches: mechanisms that promote stem cell maintenance throughout life. *Cell*.

[2] Inman J. L., Robertson C., Mott J. D., Bissell M. J. (2015). Mammary gland development: cell fate specification, stem cells and the microenvironment. *Development*.

[3] Hennighausen L., Robinson G. W. (2005). Information networks in the mammary gland. *Nature Reviews Molecular Cell Biology*.

[4] Polyak K., Kalluri R. (2010). The role of the microenvironment in mammary gland development and cancer. *Cold Spring Harbor Perspectives in Biology*.

[5] Skibinski A., Kuperwasser C. (2015). The origin of breast tumor heterogeneity. *Oncogene*.

[6] Talhouk R., Chin J., Unemori E., Werb Z., Bissell M. (1991). Proteinases of the mammary gland: developmental regulation in vivo and vectorial secretion in culture. *Development*.

[7] Hoshino K., Gardner W. U. (1967). Transplantability and life span of mammary gland during serial transplantation in mice. *Nature*.

[8] Daniel C. W., de Ome K. B., Young J. T., Blair P. B., Faulkin L. J. (1968). The in vivo life span of normal and preneoplastic mouse mammary glands: a serial transplantation study. *Proceedings of the National Academy of Sciences of the United States of America*.

[9] Kordon E., Smith G. (1998). An entire functional mammary gland may comprise the progeny from a single cell. *Development*.

[10] Pal B., Chen Y., Vaillant F. (2017). Construction of developmental lineage relationships in the mouse mammary gland by single-cell RNA profiling. *Nature Communications*.

[11] Bach K., Pensa S., Grzelak M. (2017). Differentiation dynamics of mammary epithelial cells revealed by single-cell RNA sequencing. *Nature Communications*.

[12] Keller P. J., Arendt L. M., Kuperwasser C. (2011). Stem cell maintenance of the mammary gland: it takes two. *Cell Stem Cell*.

[13] Dong Q., Wang D., Bandyopadhyay A. (2013). Mammospheres from murine mammary stem cell-enriched basal cells: clonal characteristics and repopulating potential. *Stem Cell Research*.

[14] Eirew P., Stingl J., Raouf A. (2008). A method for quantifying normal human mammary epithelial stem cells with *in vivo* regenerative ability. *Nature Medicine*.

[15] Van Keymeulen A., Rocha A. S., Ousset M. (2011). Distinct stem cells contribute to mammary gland development and maintenance. *Nature*.

[16] Shackleton M., Vaillant F., Simpson K. J. (2006). Generation of a functional mammary gland from a single stem cell. *Nature*.

[17] Tao L., van Bragt M. P. A., Laudadio E., Li Z. (2014). Lineage tracing of mammary epithelial cells using cell-type-specific cre-expressing adenoviruses. *Stem Cell Reports*.

[18] van Amerongen R., Bowman A. N., Nusse R. (2012). Developmental stage and time dictate the fate of Wnt/*β*-catenin-responsive stem cells in the mammary gland. *Cell Stem Cell*.

[19] Deome K. B., Faulkin LJ, Bern H., Blair P. (1959). Development of mammary tumors from hyperplastic alveolar nodules transplanted into gland-free mammary fat pads of female C3H mice. *Cancer Research*.

[20] Smith G. H. (1996). Experimental mammary epithelial morphogenesis in an in vivo model: evidence for distinct cellular progenitors of the ductal and lobular phenotype. *Breast Cancer Research and Treatment*.

[21] Stingl J., Eirew P., Ricketson I. (2006). Purification and unique properties of mammary epithelial stem cells. *Nature*.

[22] Badders N. M., Goel S., Clark R. J. (2009). The Wnt receptor, Lrp5, is expressed by mouse mammary stem cells and is required to maintain the basal lineage. *PLoS One*.

[23] Zeng Y. A., Nusse R. (2010). Wnt proteins are self-renewal factors for mammary stem cells and promote their long-term expansion in culture. *Cell Stem Cell*.

[24] Spike B. T., Engle D. D., Lin J. C., Cheung S. K., La J., Wahl G. M. (2012). A mammary stem cell population identified and characterized in late embryogenesis reveals similarities to human breast cancer. *Cell Stem Cell*.

[25] dos Santos C. O., Rebbeck C., Rozhkova E. (2013). Molecular hierarchy of mammary differentiation yields refined markers of mammary stem cells. *Proceedings of the National Academy of Sciences of the United States of America*.

[26] Machado H. L., Kittrell F. S., Edwards D. (2013). Separation by cell size enriches for mammary stem cell repopulation activity. *Stem Cells Translational Medicine*.

[27] Plaks V., Brenot A., Lawson D. A. (2013). *Lgr5*-expressing cells are sufficient and necessary for postnatal mammary gland organogenesis. *Cell Reports*.

[28] Prater M. D., Petit V., Alasdair Russell I. (2014). Mammary stem cells have myoepithelial cell properties. *Nature Cell Biology*.

[29] Wang D., Cai C., Dong X. (2015). Identification of multipotent mammary stem cells by protein C receptor expression. *Nature*.

[30] Alexander C. M. (2018). The Wnt signaling landscape of mammary stem cells and breast tumors. *Progress in Molecular Biology and Translational Science*.

[31] Zeng L., Cai C., Li S. (2016). Essential roles of cyclin Y-like 1 and cyclin Y in dividing Wnt-responsive mammary stem/progenitor cells. *PLoS Genetics*.

[32] Smalley M. J., Kendrick H., Sheridan J. M. (2012). Isolation of mouse mammary epithelial subpopulations: a comparison of leading methods. *Journal of Mammary Gland Biology and Neoplasia*.

[33] Kretzschmar K., Watt F. M. (2012). Lineage tracing. *Cell*.

[34] Rulands S., Simons B. D. (2016). Tracing cellular dynamics in tissue development, maintenance and disease. *Current Opinion in Cell Biology*.

[35] Carlone D. L. (2016). Identifying adult stem cells using Cre-mediated lineage tracing. *Current Protocols in Stem Cell Biology*.

[36] Rios A. C., Fu N. Y., Lindeman G. J., Visvader J. E. (2014). In situ identification of bipotent stem cells in the mammary gland. *Nature*.

[37] Visvader J. E., Stingl J. (2014). Mammary stem cells and the differentiation hierarchy: current status and perspectives. *Genes & Development*.

[38] Zhu Y., Huang Y. F., Kek C., Bulavin D. V. (2013). Apoptosis differently affects lineage tracing of Lgr5 and Bmi1 intestinal stem cell populations. *Cell Stem Cell*.

[39] Wuidart A., Ousset M., Rulands S., Simons B. D., Van Keymeulen A., Blanpain C. (2016). Quantitative lineage tracing strategies to resolve multipotency in tissue-specific stem cells. *Genes & Development*.

[40] Rios A. C., Fu N. Y., Cursons J., Lindeman G. J., Visvader J. E. (2016). The complexities and caveats of lineage tracing in the mammary gland. *Breast Cancer Research*.

[41] Rodilla V., Dasti A., Huyghe M. (2015). Luminal progenitors restrict their lineage potential during mammary gland development. *PLoS Biology*.

[42] Lilja A. M., Rodilla V., Huyghe M. (2018). Clonal analysis of Notch1-expressing cells reveals the existence of unipotent stem cells that retain long-term plasticity in the embryonic mammary gland. *Nature Cell Biology*.

[43] Sale S., Lafkas D., Artavanis-Tsakonas S. (2013). Notch2 genetic fate mapping reveals two previously unrecognized mammary epithelial lineages. *Nature Cell Biology*.

[44] Lafkas D., Rodilla V., Huyghe M., Mourao L., Kiaris H., Fre S. (2013). Notch3 marks clonogenic mammary luminal progenitor cells in vivo. *Journal of Cell Biology*.

[45] Chang T. H.-T., Kunasegaran K., Tarulli G. A., de Silva D., Voorhoeve P. M., Pietersen A. M. (2014). New insights into lineage restriction of mammary gland epithelium using parity-identified mammary epithelial cells. *Breast Cancer Research*.

[46] Wang C., Christin J. R., Oktay M. H., Guo W. (2017). Lineage-biased stem cells maintain estrogen-receptor-positive and -negative mouse mammary luminal lineages. *Cell Reports*.

[47] Sreekumar A., Toneff M. J., Toh E. (2017). WNT-mediated regulation of FOXO1 constitutes a critical axis maintaining pubertal mammary stem cell homeostasis. *Developmental Cell*.

[48] Wuidart A., Sifrim A., Fioramonti M. (2018). Early lineage segregation of multipotent embryonic mammary gland progenitors. *Nature Cell Biology*.

[49] Van Keymeulen A., Fioramonti M., Centonze A., Bouvencourt G., Achouri Y., Blanpain C. (2017). Lineage-restricted mammary stem cells sustain the development, homeostasis, and regeneration of the estrogen receptor positive lineage. *Cell Reports*.

[50] Elias S., Morgan M. A., Bikoff E. K., Robertson E. J. (2017). Long-lived unipotent Blimp1-positive luminal stem cells drive mammary gland organogenesis throughout adult life. *Nature Communications*.

[51] Scadden D. T. (2006). The stem-cell niche as an entity of action. *Nature*.

[52] Joshi P. A., Di Grappa M. A., Khokha R. (2012). Active allies: hormones, stem cells and the niche in adult mammopoiesis. *Trends in Endocrinology & Metabolism*.

[53] Mallepell S., Krust A., Chambon P., Brisken C. (2006). Paracrine signaling through the epithelial estrogen receptor *α* is required for proliferation and morphogenesis in the mammary gland. *Proceedings of the National Academy of Sciences of the United States of America*.

[54] Feng Y., Manka D., Wagner K. U., Khan S. A. (2007). Estrogen receptor-*α* expression in the mammary epithelium is required for ductal and alveolar morphogenesis in mice. *Proceedings of the National Academy of Sciences of the United States of America*.

[55] Asselin-Labat M. L., Shackleton M., Stingl J. (2006). Steroid hormone receptor status of mouse mammary stem cells. *Journal of the National Cancer Institute*.

[56] Asselin-Labat M. L., Vaillant F., Sheridan J. M. (2010). Control of mammary stem cell function by steroid hormone signalling. *Nature*.

[57] Joshi P. A., Jackson H. W., Beristain A. G. (2010). Progesterone induces adult mammary stem cell expansion. *Nature*.

[58] Lee H. J., Gallego-Ortega D., Ledger A. (2013). Progesterone drives mammary secretory differentiation via RankL-mediated induction of Elf5 in luminal progenitor cells. *Development*.

[59] Cai C., Yu Q. C., Jiang W. (2014). R-spondin1 is a novel hormone mediator for mammary stem cell self-renewal. *Genes & Development*.

[60] Pontier S. M., Muller W. J. (2008). Integrins in mammary-stem-cell biology and breast-cancer progression – a role in cancer stem cells?. *Journal of Cell Science*.

[61] Hynes R. O. (2002). Integrins: bidirectional, allosteric signaling machines. *Cell*.

[62] Taddei I., Deugnier M.-A., Faraldo M. M. (2008). *β*1 integrin deletion from the basal compartment of the mammary epithelium affects stem cells. *Nature Cell Biology*.

[63] Kessenbrock K., Dijkgraaf G. J. P., Lawson D. A. (2013). A role for matrix metalloproteinases in regulating mammary stem cell function via the Wnt signaling pathway. *Cell Stem Cell*.

[64] Mori H., Lo A. T., Inman J. L. (2013). Transmembrane/cytoplasmic, rather than catalytic, domains of Mmp14 signal to MAPK activation and mammary branching morphogenesis via binding to integrin *β*1. *Development*.

[65] Eaves C. J. (2008). Cancer stem cells: here, there, everywhere?. *Nature*.

[66] Brooks M. D., Burness M. L., Wicha M. S. (2015). Therapeutic implications of cellular heterogeneity and plasticity in breast cancer. *Cell Stem Cell*.

[67] Vassilopoulos A., Chisholm C., Lahusen T., Zheng H., Deng C. X. (2014). A critical role of CD29 and CD49f in mediating metastasis for cancer-initiating cells isolated from a Brca1-associated mouse model of breast cancer. *Oncogene*.

[68] Yang L., Tang H., Kong Y. (2015). LGR5 promotes breast cancer progression and maintains stem-like cells through activation of Wnt/*β*-catenin signaling. *Stem Cells*.

[69] Hwang-Verslues W. W., Kuo W.-H., Chang P.-H. (2009). Multiple lineages of human breast cancer stem/progenitor cells identified by profiling with stem cell markers. *PLoS One*.

[70] Vaillant F., Asselin-Labat M. L., Shackleton M., Forrest N. C., Lindeman G. J., Visvader J. E. (2008). The mammary progenitor marker CD61/*β*3 integrin identifies cancer stem cells in mouse models of mammary tumorigenesis. *Cancer Research*.

[71] Lim E., Wu D., Pal B. (2010). Transcriptome analyses of mouse and human mammary cell subpopulations reveal multiple conserved genes and pathways. *Breast Cancer Research*.

[72] Holland J. D., Klaus A., Garratt A. N., Birchmeier W. (2013). Wnt signaling in stem and cancer stem cells. *Current Opinion in Cell Biology*.

[73] Li Y., Welm B., Podsypanina K. (2003). Evidence that transgenes encoding components of the Wnt signaling pathway preferentially induce mammary cancers from progenitor cells. *Proceedings of the National Academy of Sciences of the United States of America*.

[74] Koren S., Reavie L., Couto J. P. (2015). *PIK3CA *
^H1047R^ induces multipotency and multi-lineage mammary tumours. *Nature*.

[75] van Keymeulen A., Lee M. Y., Ousset M. (2015). Reactivation of multipotency by oncogenic PIK3CA induces breast tumour heterogeneity. *Nature*.

[76] Bjerkvig R., Tysnes B. B., Aboody K. S., Najbauer J., Terzis A. J. A. (2005). The origin of the cancer stem cell: current controversies and new insights. *Nature Reviews Cancer*.

